# Influence of lung CT changes in chronic obstructive pulmonary disease (COPD) on the human lung microbiome

**DOI:** 10.1371/journal.pone.0180859

**Published:** 2017-07-13

**Authors:** Marion Engel, David Endesfelder, Brigitte Schloter-Hai, Susanne Kublik, Michael S. Granitsiotis, Piera Boschetto, Mariarita Stendardo, Imre Barta, Balazs Dome, Jean-François Deleuze, Anne Boland, Joachim Müller-Quernheim, Antje Prasse, Tobias Welte, Jens Hohlfeld, Deepak Subramanian, David Parr, Ivo Glynne Gut, Timm Greulich, Andreas Rembert Koczulla, Adam Nowinski, Dorota Gorecka, Dave Singh, Sumit Gupta, Christopher E. Brightling, Harald Hoffmann, Marion Frankenberger, Thomas P. Hofer, Dorothe Burggraf, Marion Heiss-Neumann, Loems Ziegler-Heitbrock, Michael Schloter, Wolfgang zu Castell

**Affiliations:** 1 Scientific Computing Research Unit, Institute of Computational Biology, Helmholtz Zentrum München, Neuherberg, Germany; 2 Research Unit Comparative Microbiome Analysis, Helmholtz Zentrum München, Neuherberg, Germany; 3 Department of Medical Sciences, University of Ferrara, Ferrara, Italy; 4 Department of Pathophysiology, National Koranyi Institute for TB and Pulmonology, Budapest, Hungary; 5 Centre National de Génotypage, Institut de Génomique, CEA, Evry, France; 6 Department of Pneumology, University Medical Center, Freiburg, Germany; 7 Department of Respiratory Medicine, Hannover Medical School, Hannover, Germany; 8 Member of the German Center for Lung Research (DZL), Giessen, Germany; 9 Fraunhofer Institute for Toxicology and Experimental Medicine, Hannover, Germany; 10 Department of Respiratory Medicine, University Hospitals Coventry and Warwickshire NHS Trust, Coventry, United Kingdom; 11 CNAG-CRG, Centre for Genomic Regulation, Barcelona Institute for Science and Technology, Barcelona, Spain; 12 Department of Medicine, Pulmonary and Critical Care Medicine, University Medical Center Giessen and Marburg Philipps-University, Marburg, Germany; 13 Second Department of Respiratory Medicine, National Tuberculosis and Lung Diseases Research Institute, Warsaw, Poland; 14 University of Manchester, Medicines Evaluation Unit and University Hospital of South Manchester Foundation Trust, Manchester, United Kingdom; 15 Institute for Lung Health, Department of Infection, Immunity and Inflammation, University of Leicester, Leicester, United Kingdom; 16 Institute of Microbiology and Laboratory Medicine, Synlab MVZ Gauting & IML red GmbH, Gauting, Germany; 17 CPC Comprehensive Pneumology Center, Helmholtz Zentrum München, Ludwig-Maximilians Universität und Asklepios Klinik Gauting, Munich, Germany; 18 EvA Study Center, Helmholtz Zentrum Muenchen, Gauting, Germany; 19 Department of Mathematics, Technische Universität München, Munich, Germany; University of Illinois at Urbana-Champaign, UNITED STATES

## Abstract

**Background:**

Changes in microbial community composition in the lung of patients suffering from moderate to severe COPD have been well documented. However, knowledge about specific microbiome structures in the human lung associated with CT defined abnormalities is limited.

**Methods:**

Bacterial community composition derived from brush samples from lungs of 16 patients suffering from different CT defined subtypes of COPD and 9 healthy subjects was analyzed using a cultivation independent barcoding approach applying 454-pyrosequencing of 16S rRNA gene fragment amplicons.

**Results:**

We could show that bacterial community composition in patients with changes in CT (either airway or emphysema type changes, designated as severe subtypes) was different from community composition in lungs of patients without visible changes in CT as well as from healthy subjects (designated as mild COPD subtype and control group) (PC1, P_adj_ = 0.002). Higher abundance of *Prevotella* in samples from patients with mild COPD subtype and from controls and of *Streptococcus* in the severe subtype cases mainly contributed to the separation of bacterial communities of subjects. No significant effects of treatment with inhaled glucocorticoids on bacterial community composition were detected within COPD cases with and without abnormalities in CT in PCoA. Co-occurrence analysis suggests the presence of networks of co-occurring bacteria. Four communities of positively correlated bacteria were revealed. The microbial communities can clearly be distinguished by their associations with the CT defined disease phenotype.

**Conclusion:**

Our findings indicate that CT detectable structural changes in the lung of COPD patients, which we termed severe subtypes, are associated with alterations in bacterial communities, which may induce further changes in the interaction between microbes and host cells. This might result in a changed interplay with the host immune system.

## Introduction

Chronic Obstructive Pulmonary Disease (COPD) is characterized by chronic cough, increased sputum production and dyspnoea. More than 3 million people died of COPD in 2012, approximately 6% of all deaths globally [1]. Whereas in high-income countries COPD is primarily caused by tobacco smoking, in low- and middle-income countries both indoor and outdoor air pollution play an important role in disease etiology [1].

COPD is associated with chronic pulmonary inflammation, with exacerbations and comorbidity contributing to the severity in the course of disease [2]. The key pathophysiological abnormalities in the lungs are small airway narrowing and fibrosis, emphysematous lung destruction and mucus hypersecretion [2]. COPD is a very heterogeneous disease, with clinical manifestations varying between individuals both in terms of presence and severity [3]. For some years now, high resolution computed tomography (CT) has been used to evaluate structural changes in lungs of COPD patients, enabling the identification of COPD subtypes [4, 5]. These are rather attributed to structural changes in the lung than to severity of airflow obstruction. In general, decreased lung density corresponds to increased emphysema severity [6] whereas airway wall thickening goes along with chronic bronchitis [7]. Thus, based on CT, COPD subtypes have been defined: (I) the airway dominated subtype, characterized by high percentage airway wall area and typically associated with chronic bronchitis [8], (II) the emphysema dominated subtype, coming along with low lung density, and (III) cases without changes in density and percentage wall area in CT [5]. This classification system has been used to differentiate specific features of COPD [9] and is recommended as a personalized approach of treatment for patients [10].

It has been common opinion for decades, that healthy lungs are almost free of microbes due to various mechanisms of pulmonary and mucociliary clearance [11] and cellular and humoral immunity [12, 13]. It was considered that the bacterial microbiota observed in the lung of individuals with diseases like COPD results from dysfunction of these described mechanisms [14]. Pathogenic bacteria were shown to evade or impair clearing mechanisms [15, 16], and cigarette smoke has been described to exert a deleterious effect as well [17], facilitating bacterial colonization of the lung.

With the emergence of cultivation-independent methods to study microbial diversity a more multifaceted picture started to show up, changing from a pathogen dominated view towards a perspective of a complex system with possible interactions between microbes as well as microbes with the host [18, 19]. It is now generally agreed that the establishment of stable and interacting communities of organisms can help to inhibit colonization by pathogens [20, 21]. However, changes in lung pathophysiology can shift the composition of the microbial community. In the case of different COPD subtypes, this might not only impact the emergence of single organisms like non-typeable *Haemophilus influenzae* or *Moraxella catarrhalis* [22] but might also differentially influence bacterial community composition in general. Recent studies described differences on the level of whole microbial community in lungs of patients suffering from moderate to severe COPD, compared to healthy individuals [23, 24].

In this study we addressed the question of whether COPD sub-types, defined by quantitative CT, are associated with lung microbiome changes. We postulated that differences in emphysema and airway wall thickness are major factors triggering microbial community composition. Thus, we hypothesized that differences in the lung bacterial community composition are observable (I) between COPD patients and controls or (II) between COPD patients with and without CT abnormalities. To test our hypothesis, we compared lung microbial community composition in COPD patients with or without structural lung changes detectable by quantitative CT and a healthy control group. Samples were derived from participants of the EvA (acronym for “emphysema vs. airway disease” in COPD) study [4]. Material was collected by protected bronchial brushings gained during bronchoscopy. Lower airway microbiota composition was determined using a cultivation-independent barcoding approach. Changes in bacterial community composition in lung samples obtained from our study participants were assessed both on the level of individual taxa, as well as on the level of network structures.

## Material and methods

### Study population

Sixteen male and female, Caucasian patients between the age of 48 and 74 with COPD, mainly stages 1 and 2 according to GOLD classification [25] and nine controls were selected from participants from 4 European countries of the EvA study according to following criteria: >2 μg of total DNA in combination with high percentage airway wall area or low lung density in CT for cases [4]. Table 1 summarizes information of the study participants. Participants had a substantial smoke exposure (Table 1) but all were ex-smokers at the time of sampling. Except for one case they stopped smoking at least one year before the bronchoscopy procedure. None of the patients received oral glucocorticoids, antibiotics or had an exacerbation during the preceding 2 months. Nine of the cases received inhaled glucocorticoids in combination with long-acting beta-2 agonists (LABA). Twelve of the cases showed abnormalities detected by quantitative computed tomography (QCT), with underlying values for QCT indices of emphysema (lung densitometry expressed as 15th percentile point) and airway wall geometry (percentage airway wall of the apical segment bronchus of the right upper lobe), being described in detail before [26]. In summary, cases with detectable changes in CT either showed changes characteristic for airway-type COPD with high percentage wall area (≥69.3%WA) or those typical for emphysema-type COPD, characterized by low density of the lung parenchyma (≤-925.6 Hounsfield units (HU)) (S1 Table). Four cases didn’t show CT detectable changes in the lung and were designated as mild subtypes with low percentage wall area (<69.3%WA) and normal density (>-925.6 HU).

**Table 1:** Clinical, spirometry and laboratory comparisons of patients and controls. Data presented as mean ±SD, unless otherwise indicated. Abbreviations: av. last cigarette (years): average years study participants stopped smoking before bronchoscopy procedure. BMI: body mass index. pO_2_: partial pressure of oxygen. pCO_2_: partial pressure of carbon dioxide. FEV_1_: forced expiratory volume in 1s. FVC: forced vital capacity. LABA: long-acting beta-2 agonists.

study participants	cases with changes in CT (severe subtype)	cases without changes in CT (mild subtype)	control
**number of participants in respective group**	12	4	9
**sex (% male)**	66.6	100	66.6
**av. Age (year)**	65.7±7.1	59±6.5	60±9
**never-smokers**	0	0	2
**smoking, average pack-years**	50.3±18.2	57.8±16.1	27.8±13
**av. last cigarette (year)**	9.6±9.1	3.5 ±3.1	13.6±8
**av. BMI (kg/m2**)	26.2±4.4	28.7±3.8	26.0±2.9
**GOLD classification**	GOLD1:50%; GOLD2:41.7%; GOLD3: 8.3%	GOLD1:25%; GOLD2:50%; GOLD3: 25%	-1
**combined assessment group**	A: 66.7%; B:25%; C: 8.3%	A: 75%; C: 25%	NA
**pO2**	72.1±11.6	65.7±10.8	79.9±8.7
**pCO2**	35.1±3.8	38.0±3.2	36.6±1.6
**FEV**_**1**_**, % predicted**	0.77±0.16	0.72±0.17	1.2±0.2
**FEV1/FVC, %**	59.6±9.3	59.1±5.3	81.1±3.3
**% applying inhaled glucocorticoid/LABA**	58.3	50	0

The study was approved by the local ethics and review boards at the participating centers: Ethics Committee of Ferrara (Nr. 071195 (2007)), Medical Research Council, Scientific and Research Committee (ETT-TUKEB, 22-278/2007-1018EKU), Leicestershire, Northamptonshire and Rutland MREC (08/H0402/19), Ethics Committee of the Philipps-University of Marburg (101/08, 04.07.2008) and all subjects provided written informed consent.

### Lung material

Flexible bronchoscopy was performed after mild sedation in supine position. In the right upper lobe segment bronchi S1, and its sub-segments were sampled with a protected brush (5mm diameter at bristle level, Olympus, Hamburg, Germany) via a nostril as described before [4]. Bronchial brushings were transferred into RNAprotect (Qiagen, Hilden, Germany) fluid immediately, and all material was stored at -20°C. DNA was extracted using the semi-automated Qiacube, AllPrep DNA/RNA Mini Kit (Qiagen) in the CEA DNA extraction unit (Centre National de Génotypage, Institut de Génomique, CEA, Evry, France). Quantity was determined in duplicate, using the Quant-IT kits from Life Technologies (Carlsbord, California, US). Quality of DNA was verified by ensuring its integrity in 0.5% agarose gels. Samples were selected for the microbiome study if they contained more than 2 μg of total DNA with high integrity.

### Pyrosequencing

Amplification of the V6–V9 region of the 16S rRNA gene was performed according to Timmers et al. [27]. Briefly, for PCR 16S rRNA gene targeting forward primer 926F (5´-AAACTYAAAKGAATTGACGG-3´ [28]) attached to the Roche A adapter for 454-library construction and reverse primer 630R (5´-CAKAAAGGAGGTGATCC-3´ [29]) attached to the Roche B adapter were used. For multiplexing purposes each primer included a 10-nt barcode sequence. Three independent PCRs were performed for each sample with the Fast Start High Fidelity PCR System (Roche, Mannheim, Germany) containing 200ng of DNA and 0,3% (w/v) BSA with an optimal annealing temperature of 50°C and 36 cycles. Replicate PCR reactions were pooled together and purified using QiaQuick PCR Purification Kit (Qiagen, Hilden, Germany). Additional purification was performed with Agencourt AMPure XP Beads (Beckman Coulter, Krefeld, Germany) as recommended in the 454 instructions for amplicon library preparation (Roche, Mannheim, Germany). Sequencing of the 16S rRNA gene amplicon library was performed on a Roche 454 GS FLX Titanium system Pyrosequencer as recommended in the manufacturer’s instructions (Roche, Mannheim, Germany). Resulting sequences were processed using the amplicon signal processing pipeline of the Roche software for base calling, trimming of adaptors and quality trimming with one modification in the quality filtering section, where “vfScanAllFlows” was changed from “tionly” to “false” such that 3’ trimming for the Amplicon pipeline is activated leading to fewer rejected reads.

Sequence data have been submitted to Sequence Read Archive (SRA) of NCBI (BioProject ID: PRJNA296567).

### Data analysis

Unassembled sequence reads in sff format where subjected to further quality checking using mothur v.1.29.2 [30]. Briefly, after demultiplexing of the reads and trimming them to 720 flows, sequences where subjected to denoising (PyroNoise implemented in mothur). After removal of reads shorter than 200nt or with more than seven homopolymers, sequences were aligned against the Silva v102 compatible SSU reference alignment. For further reduction of sequencing errors preclustering was applied which clusters sequences only differing in up to two nucleotides. Chimera check against the Silva database was conducted applying Chimera UCHIME, as implemented using the "chimera.uchime" command in mothur and chimeric sequences were dismissed. To account for possible contamination bias, OTUs were first clustered on 99% identity and all sequencing reads from OTUs that had a significant (P<0.05) and negative spearman correlation with amplicon concentration in one of the three library amplification runs were removed for further analysis as previously described [31]. The remaining sequencing reads were subjected to a second run of the mothur software by clustering OTUs at an identity cutoff of 95%. Subsequent classification of OTUs was performed using the RDP trainset applying a bootstrap cutoff of 80%. For all OTUs that were unclassified on genus level but classified on family level representative sequence reads were retrieved, automatically aligned using SINA [32] and imported in ARB [33]. Maximum Likelihood Treeing algorithm PhyML, embedded in ARB, was used to reconstruct phylogenetic trees of partial 16S rRNA gene sequences of selected OTUs derived from this study and of reference organisms. All OTUs that could be assigned to a genus using ARB were re-classified to the corresponding genus.

### Statistical analysis

Abundances of all OTUs that were classified on genus level were added to obtain the abundance of their corresponding genus. Genera with less than 0.01% abundance within the total number of samples were neglected. To visualize differences in community composition between COPD subtypes, PCoA analysis was performed using Bray-Curtis distances [34] on Hellinger transformed abundances. We observed a trend for differences in community composition between samples processed during summer and samples processed during winter (S1 Fig). To avoid systematic bias, reported P-values for comparisons of individual abundances, diversity and principal coordinates were corrected for seasonal effects. This was achieved by using the difference of the value under consideration and the median of this value for all samples processed during winter or summer, respectively. For the estimation of diversity in terms of species evenness and richness, we applied Pielou’s evenness [35] and the Chao richness index [48]. In addition, rarefaction curves and PERMANOVA were computed using the R package vegan (S2 Fig) [49]. To test for differences in abundances between binary meta-variables, two-sided Wilcoxon-Mann-Whitney tests were applied. P-values of individual genera and PERMANOVA analyses were adjusted for multiple testing using Benjamini-Hochberg’s method [36]. Such P-values will be referred to as P_adj_ throughout the manuscript. To construct interaction networks of genera, we used the CCREPE method [37] with pairwise Spearman’s rank correlation coefficients for relative abundances of all genera larger than zero in at least 5 samples. To obtain P-values we used 1000 random permutations and set an edge, if P<0.05 and the correlation coefficient passed a threshold of 0.5. To account for differences between samples processed during winter and summer, correlations were also computed for abundances corrected for seasonal effects and all edges that did not remain significant were removed from the initial network. Communities of correlated genera were then defined based on the resulting connected components.

## Results

After direct extraction of the DNA derived from bronchial brushings from patients suffering from COPD stages 1 and 2 according to the GOLD classification, and from healthy subjects, bacterial community composition was determined by 454 pyro-sequencing of 16S rRNA gene amplification products. Sequencing resulted in 77515 chimera free, high-quality reads. After removal of 16S rRNA sequences derived from mitochondria of epithelium cells of the lung and of reads identified as potential PCR contaminations according to [31], numbers of reads varied between 722 to 4789 reads per sample. To account for differences in the number of sequencing reads, all libraries were subsampled to 722 reads and reads were clustered on 95% sequence similarity level. This number of reads was sufficient to cover the majority of OTUs in all samples as indicated by analysis of the individual rarefaction curves (S2 Fig). Samples with less than 700 reads in total were discarded for the analysis. In total, 25 samples (9 samples from healthy subjects and 16 from cases) were used for further analysis.

### Bacterial community composition of the lung in healthy subjects and COPD patients with and without CT detectable abnormalities

**Fig 1:**
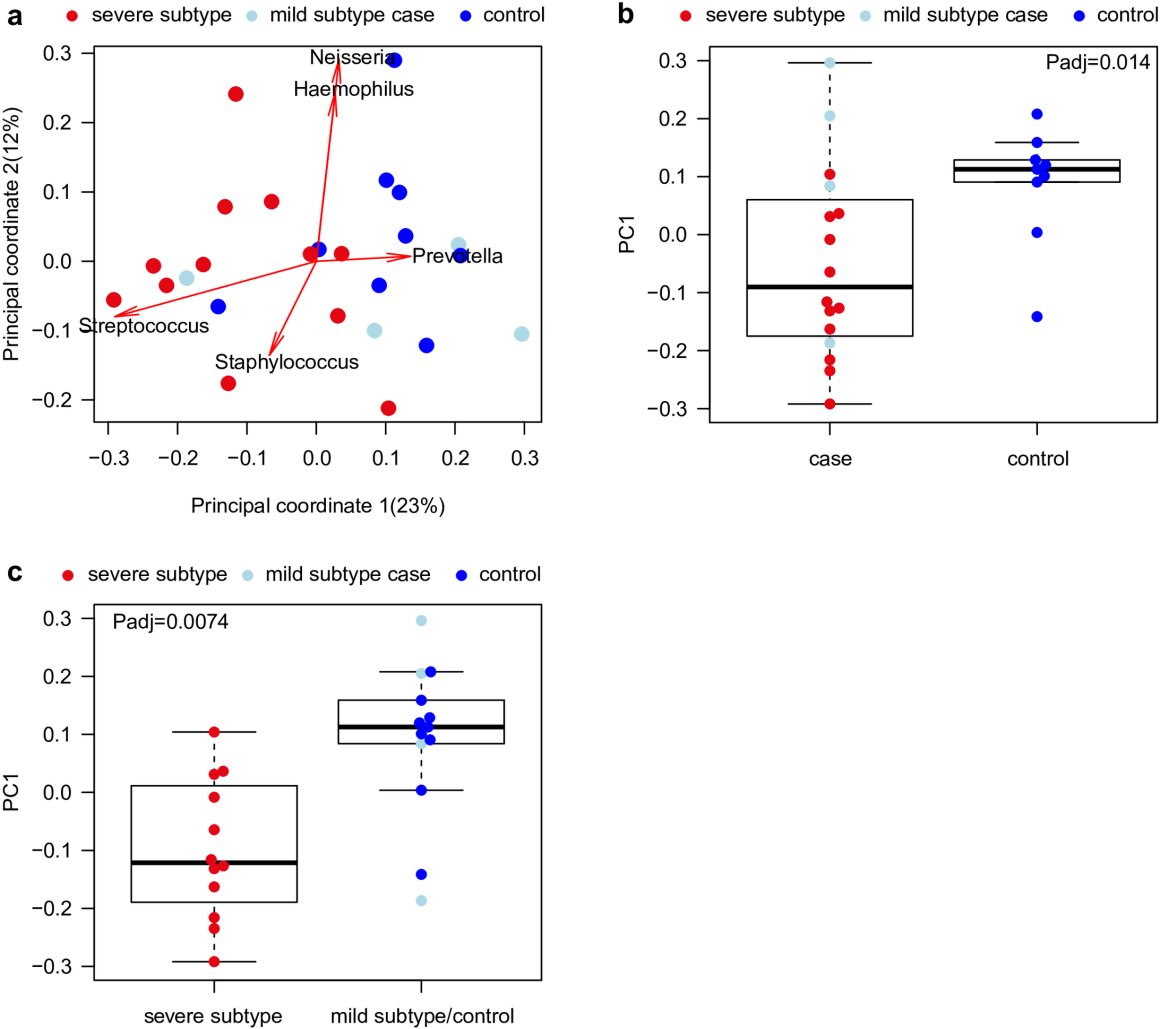
Genus level community composition for COPD patients with and without abnormalities in CT and controls based on PCoA. (**a**) Lung derived microbial community composition in severe subtype COPD patients, mild subtype cases and controls were compared based on principal coordinate 1 and 2. (**b**) Boxplot comparing principal coordinate 1 between case and control samples. (**c**) Boxplot comparing principal coordinate 1 for severe subtype and mild subtype COPD patients and control samples. P-values were computed based on two-sided Wilcoxon-Mann-Whitney tests after correction for confounding effects from samples processed during winter or summer, respectively.

Our COPD patients group comprised cases with and without CT detectable lung changes. For better readability we refer to mild COPD subtypes in the following for patients without QCT detectable changes in lungs and to severe COPD subtypes for patients with QCT detectable changes in lungs, deviating from the GOLD nomenclature of COPD patients. Such QCT detectable abnormalities were either characterized by low lung density in CT, indicating destruction of the lung parenchyma (emphysema), or airway type changes characterized by high percentage wall area. We hypothesized, that changes in lung bacterial community composition might either be observable between COPD patients and controls or between cases with compared to those without abnormalities in CTs (severe and mild subtype, respectively) and controls. To test these hypotheses, bacterial community composition in lung samples of mild and severe subtype COPD patients and controls was analyzed based on Principal Coordinate Analysis (PCoA) (Fig 1A). While the latter indicated separation of communities of controls and patients (principal coordinate (PC) 1, P_adj_ = 0.014) (Fig 1B), no significant differences were observed based on PERMANOVA analysis (P_adj_ = 0.11). Since changes in QCT reflect structural changes of the lung and since these changes may be more important to microbial composition than air flow parameters, we compared microbial patterns in cases with CT changes and individuals with normal lung structure (cases without CT changes and controls). This analysis showed, that the main difference in community composition was observed between severe versus mild subtype cases and controls in the first principal coordinate (PC1, P_adj_ = 0.0074) (Fig 1C) and in PERMANOVA (P_adj_ = 0.006) analyses. Moreover, bacterial communities derived from lungs of mild subtype COPD patients closely resembled those of controls (P = 0.60). Furthermore, communities derived from severe subtype COPD patients could be separated from controls (P_adj_ = 0.0048) (S4 Fig). The focus of the following analyses was therefore on the comparisons between COPD patients showing visible changes in CT designated as severe subtype cases with those without changes and controls (designated as mild subtype and control group).

**Fig 2:**
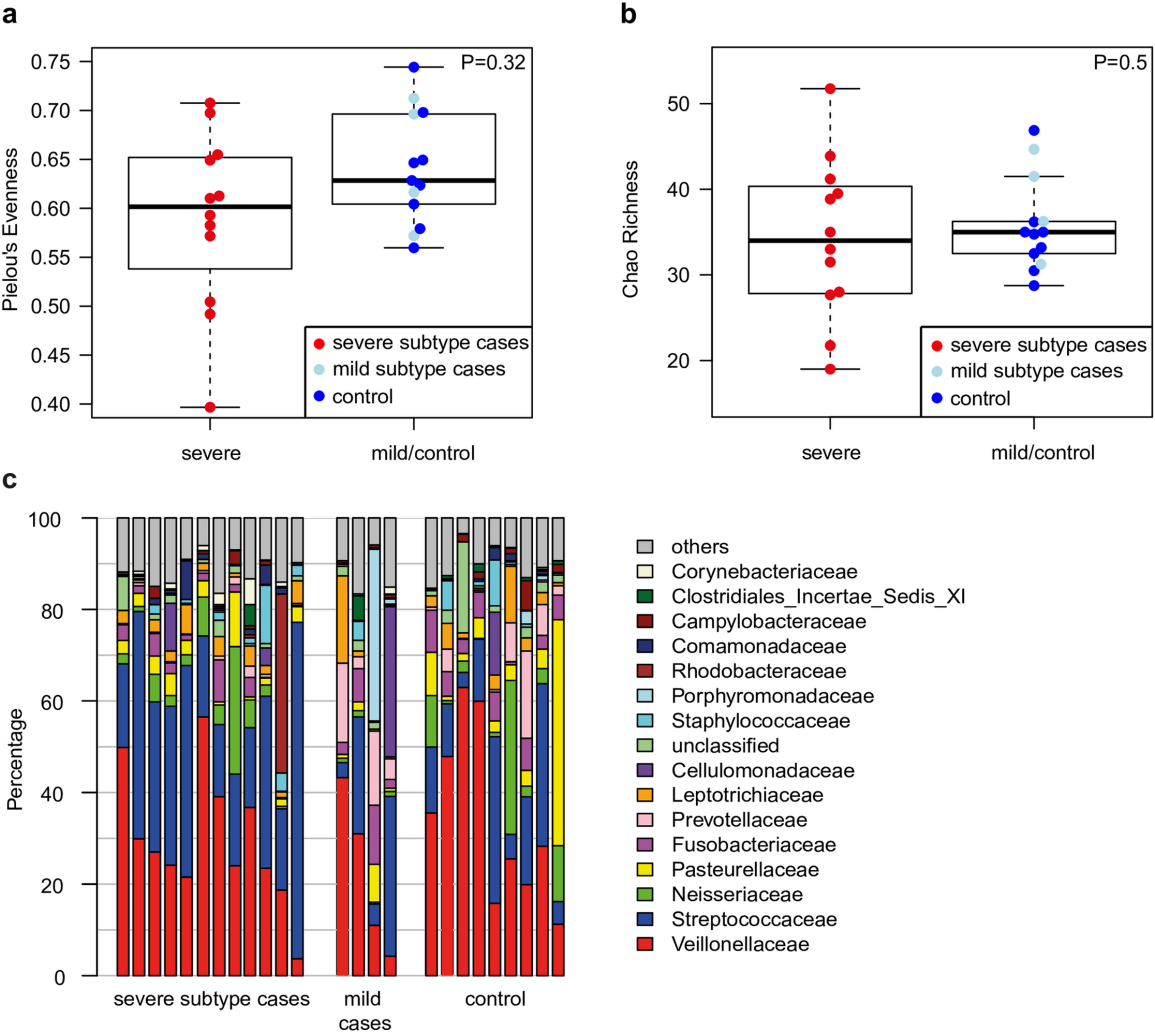
Diversity of microbial communities derived from lungs of severe subtype cases and mild subtype and control individuals. (**a**) Pielou’s evenness and (**b**) Chao richness of microbial communities on genus level for severe vs mild/control samples. P-values were computed based on two-sided Wilcoxon-Mann-Whitney tests after correction for confounding effects from samples processed during winter or summer, respectively. (**c**) Phylogenetic community composition based on 16S rRNA gene fragment sequences in bronchial brushing samples. Bars represent the relative abundance of the most abundant families (>5% in one sample). The remaining families are subsumed as others.

We hypothesized, that changes in the structure of the lung might coincide with differences in diversity or richness of harbored bacterial communities. Thus, evenness and richness of community compositions was compared between severe vs mild subtype cases and controls. No significant differences were observed between groups (P = 0.32 and 0.5, respectively) (Fig 2A and 2B). The composition of bacterial communities on family level, showed a large variability between the individual subjects (Fig 2C). However, representatives of *Veillonellaceae* and *Streptococcaceae* were dominating the microbial communities in most samples, summing up to more than 50% of bacterial families present in the majority of samples of cases as well as controls. Frequently detected bacterial families, although less dominant, were *Neisseriaceae*, *Pasteurellaceae and Fusobacteriaceae* (Fig 2C).

### Differences in individual genera between severe vs mild subtype cases and controls

**Fig 3:**
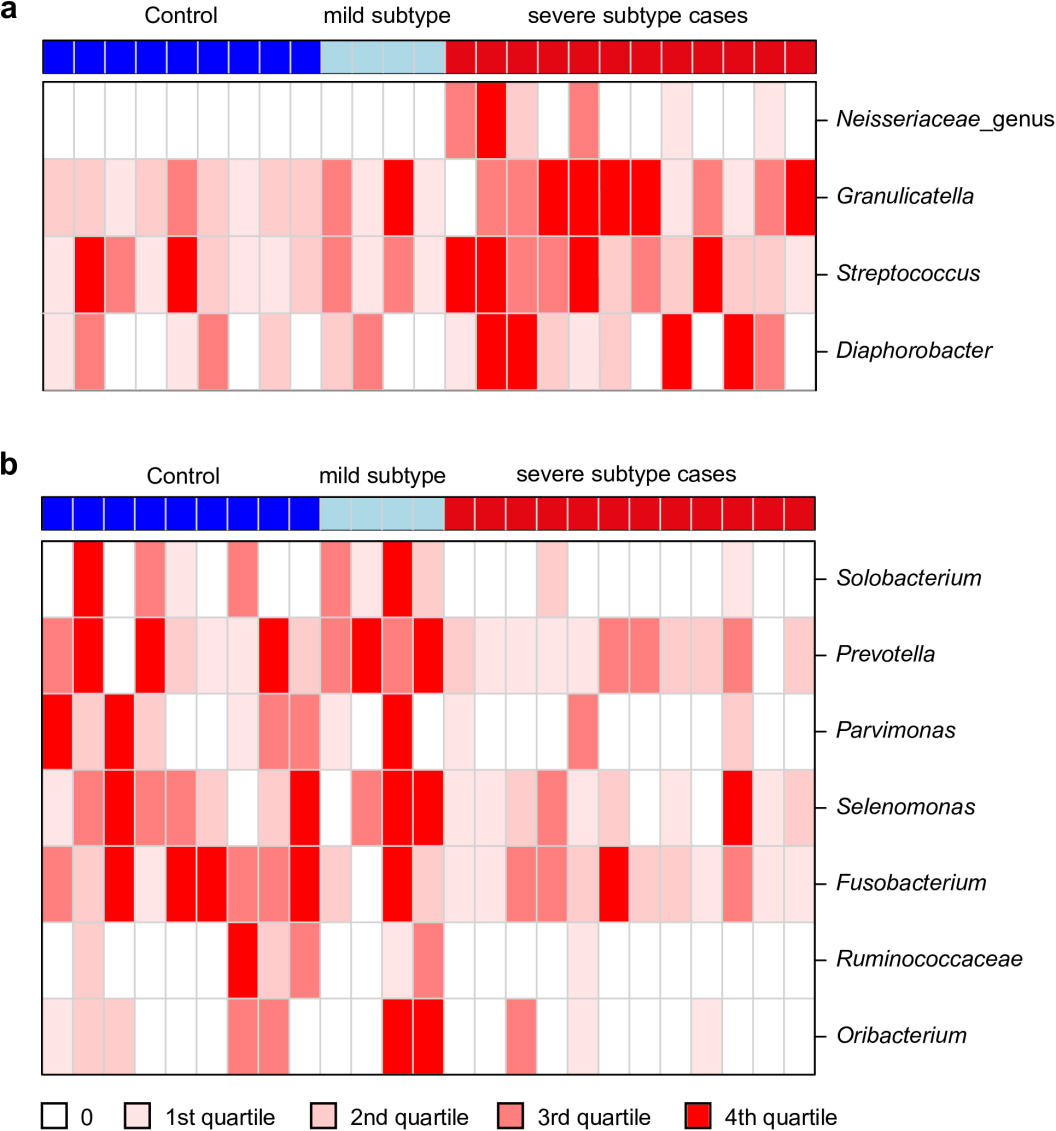
Heatmap of genera showing significantly different abundances between severe versus mild subtype cases and controls. Genera showing significantly increased (**a**) or decreased (**b**) abundances in severe vs mild subtype cases and controls. Colors at the top represent COPD subtypes and controls. For each genus, samples were colored based on quartiles of non-zero abundances from light red to dark red. All Samples for which a genus was not present were colored in white.

To identify genera which contribute to the separation of severe versus mild subtype cases and the control group, all genera were screened for significant differences between the two groups of individuals (Fig 3 and S5 Fig). *Streptococcus* (P = 0.03) (Fig 1A), *Granulicatella* (P = 0.018), a *Neisseriaceae* genus (P = 0.002), and *Diaphorobacter* (P = 0.02) showed significantly higher abundance in severe subtype COPD cases (Fig 3A and S5 Fig). In the mild subtype and control group significantly higher abundances of genera *Prevotella* (P = 0.008) (Fig 1), *Solobacterium* (P = 0.018), *Parvimonas* (P = 0.0038), *Selenomonas* (P = 0.013), *Fusobacterium* (P = 0.014), *Oribacterium* (P = 0.042), and a *Ruminococcaceae* genus (P = 0.014) were contributing to the observed differences between the groups (Fig 3B and S5 Fig). However, after multiple testing corrections no significant associations remained. Thus, above described findings should be considered as trends, which have to be confirmed in further studies.

### Influence of medication on microbial community composition in the lung of COPD patients

58% of the severe subtype COPD patients and 50% of the mild subtype COPD patients received Glucocorticoid/LABA (GC) medication by inhalation (Table 1). We hypothesized that GC medication might exert an effect on bacterial community composition. However, no significant effects of GC treatment on bacterial community composition were detected for cases with and those without GC treatment in PCoA (P = 0.19) (S3 Fig). Additionally, we tested for an influence of GC treatment on the abundance of individual genera (S6 Fig). Members of *Parvimonas* showed lower abundances in patients receiving GC treatment (P = 0.0071). For representatives of *Solobacterium* (P = 0.054) and *Fusobacterium* (P = 0.065) a slight trend towards reduced abundance in samples derived from GC treated patients was observable, but is missing significance. This influence of GC treatment on individual genera might also have contributed to the observed differences for the respective genera in the comparison between severe versus the mild subtype cases and control group (S5 Fig).

### Bacterial networks in the lung

**Fig 4:**
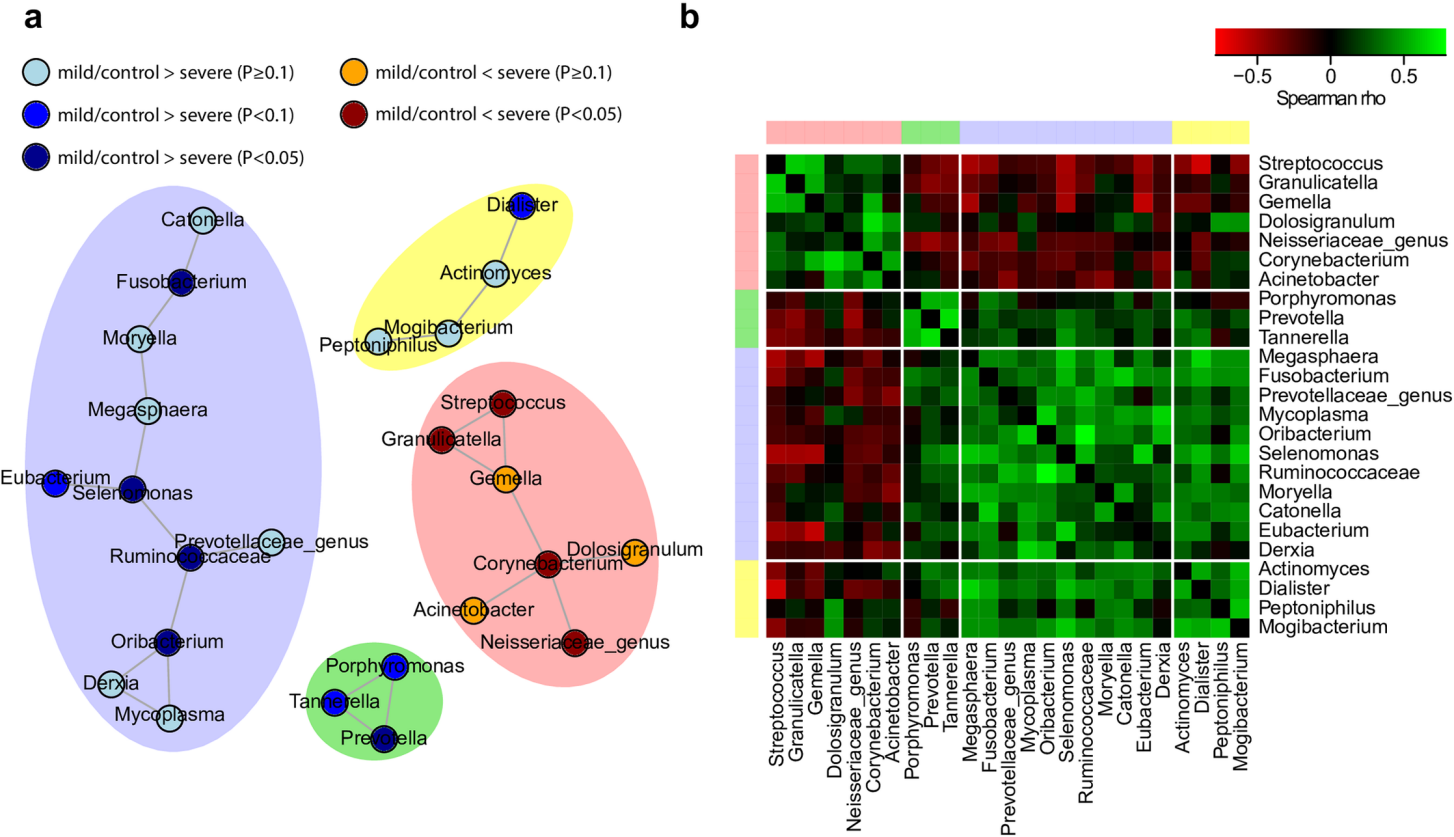
Co-occurrence analysis of bacterial genera. (**a**) Microbial correlation network of bacterial genera as a surrogate for bacterial interaction. Each node represents one microbial genus and shading colors (blue, red, green, yellow) highlight microbial communities. Coloring of nodes indicates differences between severe subtype versus mild subtype cases and controls (red colors: higher average abundance in severe subtype cases; blue colors: higher average abundance in mild subtype cases and controls). Communities containing ≤2 genera were not shown. (**b**) Heatmap showing spearman correlation coefficients between bacterial genera from the four microbial communities. Colors at the upper and left side of the figure indicate the community affiliation of respective genera.

On basis of the observed differences in community composition between severe vs mild COPD subtypes and controls, we aimed at assessing associations of bacteria in the lung. Particularly, we were interested how differences in abundances between severe versus mild subtype cases and controls influence associations within microbiota. To address this question, a microbial correlation network was estimated based on relative abundances of bacterial genera. This approach revealed four communities of positively correlated bacteria (Fig 4A).

The microbial communities can clearly be distinguished by their associations with disease phenotype. While all genera in three of the communities (marked in blue, green and yellow) showed a trend for increased abundances in the mild subtype cases and controls, all genera in the fourth community showed the opposite trend (red community). Among the communities with increased abundances in individuals of the mild subtype and control group one small community (marked in green) consisted of members of *Bacteroidetes* (S7 Fig). It included *Prevotella* which was indicative for the separation of groups in PCoA (Fig 1A). The largest community (blue) harbored mainly representatives of *Firmicutes* beside genera of *Bacteroidetes*, *Tenericutes*, *Proteobacteria* and *Fusobacteria* (S7 Fig). Many of the genera showed significantly increased abundances in the mild subtype cases and controls compared to severe subtype cases (S5 Fig). In contrast, the cluster marked in red, harbored genera predominantly detected in severe subtype cases (Fig 4A). This cluster is composed of members of *Firmicutes*, *Actinobacteria* and *Proteobacteria* (S7 Fig) and included the genus *Streptococcus* which was indicative for the separation of groups in PCoA (Fig 1A). Overall, clustering of genera into communities indicated that co-occurrence relationships were strongly influenced by differences in microbial abundances between severe subtype versus mild subtype cases and controls. Different structural states of the lung favored different clusters of genera. Further comparison of correlation patterns between community members reveals co-exclusion relationships between communities containing genera that have increased abundances in severe subtype cases and the community containing genera that have increased abundances in the mild subtype and controls (Fig 4B).

## Discussion

The current microbiota analysis was performed on samples obtained from the EvA study [4]. This study defined subtypes of COPD using CT image analysis of lung density for determination of the degree of emphysema and airway wall thickness as a measure of bronchitis. We were interested in early changes in bacterial communities during disease development in COPD, with special emphasis on interrelation of COPD subtypes with and without abnormalities in CT and changes in microbial community composition.

### Microbial community composition in the lung of COPD patients

Differences in bacterial community composition of the lung were observed between patients with severe subtypes of COPD compared to that of controls. However, bacterial communities derived from lungs of mild subtype COPD patients clustered mainly with those derived from healthy controls. Several studies reported contamination of sputum as a measure for lung microbial communities with upper airway microbiota, and thus, higher diversity in these samples or even day to day variation in absence of clinical changes can be expected [38, 39]. In our study protected specimen brushes introduced through the nose were used for sampling. This sampling technique not only minimizes contamination of lung derived samples with bacteria from the upper airways, but provides samples being derived from the interface of human and environment and thus targets those organisms which are in tight interplay with the human cells and immune system. In our study sampling was performed in the right upper lobe. Dickson et al. [40] could show that right upper lobe microbiota more closely resembled that of the upper respiratory tract, than that from more distant lung regions like middle lobe and lingual. Thus, our detection of typical representative genera of the upper respiratory tract like *Veillonella*, *Oribacterium* or *Catonella* [41] support their findings [40]. For patients with severe lung indispositions partly accompanied by exacerbations, differences in microbial community composition have been described before: in a study including 5 COPD patients and 11 cases with asthma, bacterial communities clustered together but differed in composition from communities of healthy controls [24]. However, COPD patients examined in this study have a much lower average percent of FEV1 predicted value (51,2%) [24] compared to participants of our study (Table 1). Also in another study composition of microbial community derived from lungs of patients suffering from moderate to severe COPD differed from that of the control group [23]. Sze and coworkers could show an increased diversity and significantly different community structures of bacteria derived from lung tissue of patients suffering from COPD GOLD stage 4 compared to controls [42]. However, none of the studies mentioned above, focused on the associations of COPD subtypes based on CT detectable changes in lungs of affected subjects with bacterial community composition, as performed in our study. Detection of shifts in community composition of COPD cases with detected abnormalities in CT in our study suggests that changes in microbial colonization of the lungs of COPD patients already occur early, before meaningful impairment of the health status starts. Also Sze et al. [43] were able to detect early changes by a decreased abundance of GD1-positive *Lactobacillus* in lungs of GOLD stage 1 and 2 COPD patients compared to controls, although on genus level *Lactobacillus* showed no differences between groups. However, changes like this are not detectable in studies based on 16S rRNA gene fragment analyses alone, given the missing resolution of 16S rRNA genes to strain level. In lungs of COPD GOLD stage 4 patients a shift to an increased abundance towards *Proteobacteria* could be detected within harbored microbial communities [44, 45]. In our severe subtype cases, a trend in this direction was observed with the higher abundance of *Diaphorobacter* or *Neisseriaceae* genus (Fig 3) contributing to the trend. Moreover, for cases with detected abnormalities in CT the shift towards lower abundance of *Bacteroidales* [24] and higher prevalence of *Lactobacillales* [23] detected in lung derived communities of GOLD stage 2 and 3 COPD cases was confirmed by data from our study. However, comparisons among different studies have to be dealt with caution, since different regions of the 16S rRNA gene as well as different sequencing platforms applied were shown to lead to slight differences in detected community composition [46, 47].

### Bacterial community structure and abnormalities in lung as detected by CT

Data from our study revealed differences in the composition of the lung microbiome of patients suffering from subtypes of COPD, that appear to correspond to CT-detectable changes like percentage airway wall area or development of emphysema in the diseased lung. It has been shown before, that these CT detectable changes involve physiological changes in the lung. In lungs of COPD patients with chronic bronchitis (cases with increased percentage airway wall area) reduced airway surface liquid leads to inefficient mucus clearance by reduced action of cilia of epithelial cells. This results in increased mucus adhesion and thus facilitates bacterial infections [48]. As a consequence of higher viscosity of mucus, gas diffusion is hampered. Also during emphysematous lung states gas diffusion is impeded, due to trapping of air. We hypothesize, that this might be an explanation for the observation of the increased abundance of typical bacterial genera like *Neisseria* in our study, that need elevated levels of CO_2_ for growth and are commensals of mucous membranes [49].

We hypothesize, that *Prevotella* may influence community composition and inflammation in the lung by exhibiting anti-inflammatory properties in mixed bacterial communities. Several different *Prevotella* species have been shown to reduce pro-inflammatory cytokine production induced by other Gram negative bacteria in dendritic cells [50]. Similar effects have been demonstrated in mouse models with an intrinsic toleration of *Prevotella* by the respiratory immune system due to only weak induction of airway inflammation [51]. Following this line the reduction of *Prevotella* in COPD disease might favour inflammation due to the absence of the potentially immunosuppressive *Prevotella* species, finally promoting wall thickening or emphysema. In contrast to findings using brush samples from severe subtypes of COPD cases in this study, and in lung specimens of explanted lungs of GOLD 4 COPD patients [52], Segal et al [53] described a correlation of *Prevotella* abundance with enhanced BAL inflammatory cells and eNO in asymptomatic smoking and non-smoking individuals. Pathogenicity of *Prevotella*, in particular *P*. *intermedia* has been described before for oral samples [54]. It could be shown, that for these pathogenic *Prevotella* the potential of invasion in epithelial cells is an important virulence factor [55]. However, this ability is obviously strain specific [55, 56]. Insight into the properties of *Prevotella* detected in higher abundance in the mild subtype and control group in this study would need further research with a good resolution of *Prevotella* to strain level and a metagenomics approach to allow conclusions about functional characteristics of *Prevotella* strains present in the lung.

### Influence of medication on microbial community composition in lung of cases

Some of our patients (9 out of 16) applied inhaled glucocorticoids (GC), all of them in combination with long-acting beta-agonists (LABA) and 70% also with long-acting muscarinic antagonists (LAMA). No significant effects of GC treatment on overall bacterial community composition could be detected for cases with and without GC treatment in PCoA, but a possible, slight impact on few single genera was observed. As it has long been shown that glucocorticoids influence antimicrobial activity of macrophages [57] and impact the extent of inflammation [58] the application of inhaled glucocorticoids can impact microbial community composition. In addition LABA, which is applied to cause bronchodilation, modifies the lung by inhibition of airway smooth-muscle cell proliferation and inflammatory mediator release, as well as stimulation of mucociliary transport [59]. In contrast to our findings, an earlier study [23] reported separation of bacterial communities of GOLD 2 and 3 COPD patients using inhaled corticosteroid from that of non-users based on their composition. The contrasting response pattern in our study could be related to the fact that users of inhaled corticosteroids were harbored in all COPD subtypes present in this study. It could already be shown in a study concerning the impact of combined corticosteroid and LABA treatment in patients suffering from different COPD subtypes, that treatment differentially exerts effects in sub-types of COPD patients. Only low improvements were observed during treatment of emphysema-type patients (characterized by low lung density in CT, included in our severe subtype, S1 Table) and highest improvements for the obstruction dominant subgroup (included in our severe subtype with high percentage wall area, S1 Table) [60]. Mild subtypes showed intermediate improvements [60]. Thus, given the heterogeneity of our study group, differential effects of GC on individuals can also be expected in our study. Only few bacterial genera were affected by glucocorticoid/ LABA medication throughout all these subtypes in our study. However, we cannot exclude that weak differences were masked due to small group sizes of 9 patients compared to 7 patients without GC treatment, resulting in a low power of resolution.

### Network structure

In our study co-occurrence analysis revealed the presence of clusters of co-occurring bacteria in the lungs of study participants, strongly influenced by differences in microbial abundances between severe versus mild subtype cases and controls. It is well accepted that composition of bacterial communities is influenced by positive (this might be mutualism or commensalism) as well as negative relationships (i.e. parasitism, competition) between contributing microorganisms in several organs of the human body [37]. The lung is a niche, where bacteria face limited supply of nutrients, influence of the host immune system, oxidative stress, as well as the clearance mechanisms of the host. Thus, interactions between bacteria can be expected, enabling and facilitating the colonization of the lung. Co-occurrence analysis revealed the presence of a large cluster of co-occurring bacteria showing higher abundances in the lungs of mild subtype cases and controls (Fig 4A, blue). In this cluster *Fusobacterium* is included, which was described before as part of the lung core microbiome [61]. *Fusobacterium* enables adherence between different sorts of cells of human and bacterial origin, which otherwise would not be able to interact [62]. This ability might help during colonization of the lung. On the one hand, various subspecies of this organism show the ability to effectively adhere to human epithelial cells [63, 64]. On the other hand, a high capability to form co-aggregates with diverse Gram+ and Gram- bacterial species in co-aggregation tests have been shown [65]. Thus, it might be hypothesized, that representatives of *Fusobacterium* act as interconnectors between human and bacterial cells in the lung, too. Finally, *Fusobacterium* is also supporting the growth of anaerobic organisms in oxygenated environments [66, 67]. This ability might be of increased importance in an aerated habitat like the lung which might promote the observed co-occurrence of *Fusobacterium* with representatives of *Lachnospiraceae* genera *Catonella* and *Moryella*, which have been described to be strictly anaerobic [68].

Another cluster of associated bacteria harbours *Porphyromonas* and *Prevotella* (Fig 4A, associated bacteria marked in green). For these species the ability to form heterotrophic biofilms by co-aggregation has been demonstrated, showing an intra-species variability in the co-aggregation of the partners [69]. During co-aggregations an increase in biomass of participating partners was detected [69]. This ability was suggested to promote persistence of both species in the oral habitat. Detected co-occurrence of these two genera in our study may in some way hint to similar capabilities of involved species. We hypothesize that also in the lung the ability to co-aggregate offers advantages for involved partners to compete. However, further studies using metagenomics approaches and single cell genomics will be needed to allow conclusions about functional traits and to go beyond the 16S rRNA gene fragment based solely associative findings of this study.

### Limitations of the study

Several groups reported specific bacterial DNA contamination of extraction and sequencing kits [70], reagents [71] or solutions [72] in 16S rRNA gene targeting next generation as well as metagenomic shotgun sequencing studies [73]. Different lots of an extraction kit harbored the same predominantly contaminating genera, but differed in genera detected in low prevalence [70]. The influence of contamination on high bacterial biomass containing samples has been described to be negligible. However, low bacterial biomass samples are prone to be influenced by bacterial contamination of reagents and kits [70–72]. In non-immunocompromised individuals the lung is a niche with low bacterial load. In this study negative controls were not included. Therefore, reads from contaminating bacteria were probably generated in our sequencing runs. In the study of Glassing et al. [70] more than 80 genera with far more than 100 tentative species have been detected as contaminants of different kits, most of them in low abundance. Moreover, the study of Segal et al. [72] described reads with similarity to *Streptococcus thermophilus* as contaminants, whereas *Streptococcus mitis* affiliated reads were detected as part of the microbial community harbored in supraglottic and lung samples. Thus, extracting all contaminating genera present in contaminant databases is no option, as contamination is very specific to the kits used as well as species or even strain specific. To account for this problem, we tested our sequencing reads according to the method described by Jervis-Bardy et al. [31]. These authors could show that relative abundance of OTUs identified as potential reagent contamination showed a strong inverse correlation with amplicon concentration allowing for objective removal. Adaption of their method to our sequencing evaluation workflow resulted in the detection of several OTUs as potential contaminants and removal of 2512 corresponding reads prior to analyses. Although it cannot be fully excluded that a subset of non-contaminating OTUs by chance show the same inverse correlation behavior as contaminating OTUs and is thus false positive, it is an appropriate method to account for contamination within our context. Additionally to exclude potential low abundant contamination of samples in our study, which might not be detectable with above described method, we neglected genera with less than 0.01% abundance within the total number of samples.

The restricted number of subjects per COPD subtype (12 for the severe subtype and four for the mild subtype) hindered the statistically valid detection of differences between COPD subtypes at the genus level. After rigorously correcting for multiple testing, no significant associations remained for subtype discriminating genera. Thus, described findings should be considered as trends, which have to be confirmed in further studies. Some of our findings hint towards differences in bacterial community composition in the lung, comparing subjects with emphysema and airway dominated severe COPD subtypes. However, with subject numbers as low as 4, the statistical power for presentation of results at level of significance was too low. Thus, potential differences have to be elucidated in larger studies with more balanced numbers of samples per COPD subgroup.

Finally, with the approach used, based on the analysis of 16S rRNA gene fragments it is not possible to provide data on the functional traits of the bacterial genera being present in the communities. Moreover, resolution to strain level is not possible, thus requiring validation of our findings based on studies applying meta- and single-cell genomic approaches as well as on determination of host derived effectors of the innate, humoral immune system.

## Conclusion

This study revealed changes in lung microbial community composition in GOLD stage 1 and 2 COPD cases with abnormalities in CT. We could show that lung community composition in COPD patients without abnormalities in CT resembles that of the control group, whereas, communities of patients with abnormalities in CT were significantly different. Our approach using community detection in association networks of bacteria hints to the presence of different communities of associated bacteria in the lung.

Our results suggest the presence of networks of associated bacteria, which are shifting, depending on changes in the lung. These shifts might lead to a dysbiosis in the lung with increasing severity of the disease which, at the end, might then result in altered susceptibility to exacerbation events, as well as responsiveness towards medical treatment.

Larger group sizes with a higher depth of sequencing will allow confirmation of our trends. Analyses of samples applying sequencing techniques that produce higher read lengths like PacBio [74] will allow getting a better phylogenetic resolution of our initial findings and enable deeper insights into community composition. Also a simultaneous acquisition of individual host derived factors, like inflammatory cells, inflammatory mediators or protease/anti-protease activity together with microbial community analyses is desirable. This would allow conclusions on immunological processes in the host and enable getting a deeper insight into interactions between innate and adaptive inflammatory immune responses on the one hand and microbial community composition on the other hand, both steering changes during COPD progression in lung. Finally, detection of spatial arrangements of bacteria in relation to sites of inflammation in the lung would be eligible to allow conclusions about interference of bacteria with the lung epithelium.

## Supporting information

S1 Table Clinical, spirometry and laboratory comparisons of patients and controls. Data presented as mean ±SD, unless otherwise indicated. Abbreviations: av. last cigarette (years): average years study participants stopped smoking before bronchoscopy procedure. BMI: body mass index. pO_2_: partial pressure of oxygen. pCO_2_: partial pressure of carbon dioxide. FEV_1_: forced expiratory volume in 1s. FVC: forced vital capacity. LABA: long-acting beta-2 agonists.
(DOC)

S1 Fig Genus level community composition for samples processed during summer and winter months based on PCoA. (a) Lung derived microbial community composition for samples processed during summer and winter months. (b) Boxplot comparing principal coordinate 1 between samples processed during summer and winter. The P-value was computed based on a two-sided Wilcoxon-Mann-Whitney test.
(PDF)

S2 Fig Rarefaction plot of COPD subtypes for OTUs clustered on 95% similarity level. All samples were subsampled to 722 reads. Colors of the rarefaction curves indicate the sample types. The number of reads is shown on the x-axis and the corresponding number of OTUs on the y-axis.
(PDF)

S3 Fig Genus level community composition for COPD patients with or without Glucocorticoid treatment. (**a**) Lung derived microbial community composition for COPD patients with and without Glucocorticoid treatment. (**b**) Boxplot comparing principal coordinate 1 between COPD patients with and without Glucocorticoid treatment. The P-value was computed based on a two-sided Wilcoxon-Mann-Whitney test.
(PDF)

S4 Fig Comparison of principal coordinates between severe COPD subtypes and controls. Boxplots comparing principal coordinate 1 between severe subtype cases and controls P-value was computed based on two-sided Wilcoxon-Mann-Whitney tests after correction for confounding effects from samples processed during winter or summer months, respectively.
(PDF)

S5 Fig Genera with significantly different abundances between severe subtype cases and mild subtype and control individuals. Boxplots show Hellinger transformed abundances for all genera that had significantly different abundances (P<0.05) in severe subtype versus mild subtype cases and controls. P-values were computed based on two-sided Wilcoxon-Mann-Whitney tests after correction for confounding effects from samples processed during winter or summer months, respectively.
(PDF)

S6 Fig Genera with significantly different abundances for Glucocorticoid treatment. Boxplots show Hellinger transformed abundances for all genera that had significantly different abundances (P<0.05) in individuals with Glucocorticoid treatment compared to individuals without Glucocorticoid treatment. P-values were computed based on two-sided Wilcoxon-Mann-Whitney tests after correction for confounding effects from samples processed during winter or summer months, respectively.
(PDF)

S7 Fig Phylum level composition of co-occurrence networks from bacterial genera. Colors of nodes represent the different phyla and shading colors indicate microbial communities.
(PDF)

## Abbreviations

OTU: Operational Taxonomic Unit
16S rRNA: 16S ribosomal RNA
COPD: Chronic Obstructive Pulmonary Disease
PCoA: Principal coordinates analysis
SD: Standard deviation
nt: nucleotide
HU: Hounsfield units
WA: Percentage wall area
