# Supplementary material for: Influence of lung CT changes in chronic obstructive pulmonary disease (COPD) on the human lung microbiome
Source: PLoS One. 2017 Jul 13;12(7):e0180859. doi: 10.1371/journal.pone.0180859 (PMC5509234; doi:10.1371/journal.pone.0180859)
Supplement: S1 Table — Data presented as mean ±SD, unless otherwise indicated. Abbreviations: av. last cigarette (years): average years study participants stopped smoking before bronchoscopy procedure. BMI: body mass index. pO2: partial pressure of oxygen. pCO2: partial pressure of carbon dioxide. FEV1: forced expiratory volume in 1s. FVC: forced vital capacity. LABA: long-acting beta-2 agonists. (DOC) [file pone.0180859.s001.doc]

| **sub-type** | airway type COPD | airway type  COPD | emphysema type COPD | mild | control |
| --- | --- | --- | --- | --- | --- |
| **number of participants in respective group** | 3 | 5 | 4 | 4 | 9 |
| **sex (% male)** | 66.6 | 80 | 50 | 100 | 66.6 |
| **av. Age (year)** | 70.6±4.9 | 60±6.9 | 69±3 | 59±6.5 | 60±9 |
| **never-smokers** | 0 | 0 | 0 | 0 | 2 |
| **smoking, average pack-years** | 50.4±20.2 | 54.6± 17.1 | 44.9±21.8 | 57.8±16.1 | 27.8±13 |
| **av. last cigarette (year)** | 13.7±8.4 | 3.8±2.2 | 14±12 | 3.5 ±3.1 | 13.6±8 |
| **av. BMI (kg/m2**) | 28.4±5 | 26.2±3.7 | 24.5±5.2 | 28.7±3.8 | 26.0±2.9 |
| **GOLD classification** | 1;2;2 | 1;1;1;2;2 | 1;1;2;3 | 1;2;2;3 | -1 |
| **combined assessment group** | B;B;B | A | A;A;A,C | A;A,A;C | NA |
| **pO2** | 81.4±10.6 | 74.3±11.1 | 62.5±6.2 | 65.7±10.8 | 79.9±8.7 |
| **pCO2** | 35.9±4.4 | 39.3±3.7 | 37.1±3.6 | 38.0±3.2 | 36.6±1.6 |
| **FEV_1_, % predicted** | 0.75±0.11 | 0.8±0.17 | 0.72±0.18 | 0.72±0.17 | 1.2±0.2 |
| **FEV1/FVC, %** | 62.5±7.2 | 61.9±10.3 | 54.5±9.6 | 59.1±5.3 | 81.1±3.3 |
| **% applying inhaled glucocorticoid/LABA** | 66.6 | 40 | 75 | 50 | 0 |
| **av. density from lung density histogram** | NA | -911.2±5.2 | -946.5±19.1 | -916.8±7.0 | NA |
| **% av. wall area** | 72.6±3.0 | 74.3±3.1 | 53.4 ±2.81 | 56.3±1.3 | NA |
