# Supplementary material for: Influence of lung CT changes in chronic obstructive pulmonary disease (COPD) on the human lung microbiome
Source: PLoS One. 2017 Jul 13;12(7):e0180859. doi: 10.1371/journal.pone.0180859 (PMC5509234; doi:10.1371/journal.pone.0180859)
Supplement: S1 Fig — (a) Lung derived microbial community composition for samples processed during summer and winter months. (b) Boxplot comparing principal coordinate 1 between samples processed during summer and winter. The P-value was computed based on a two-sided Wilcoxon-Mann-Whitney test. (PDF) [file pone.0180859.s002.pdf]

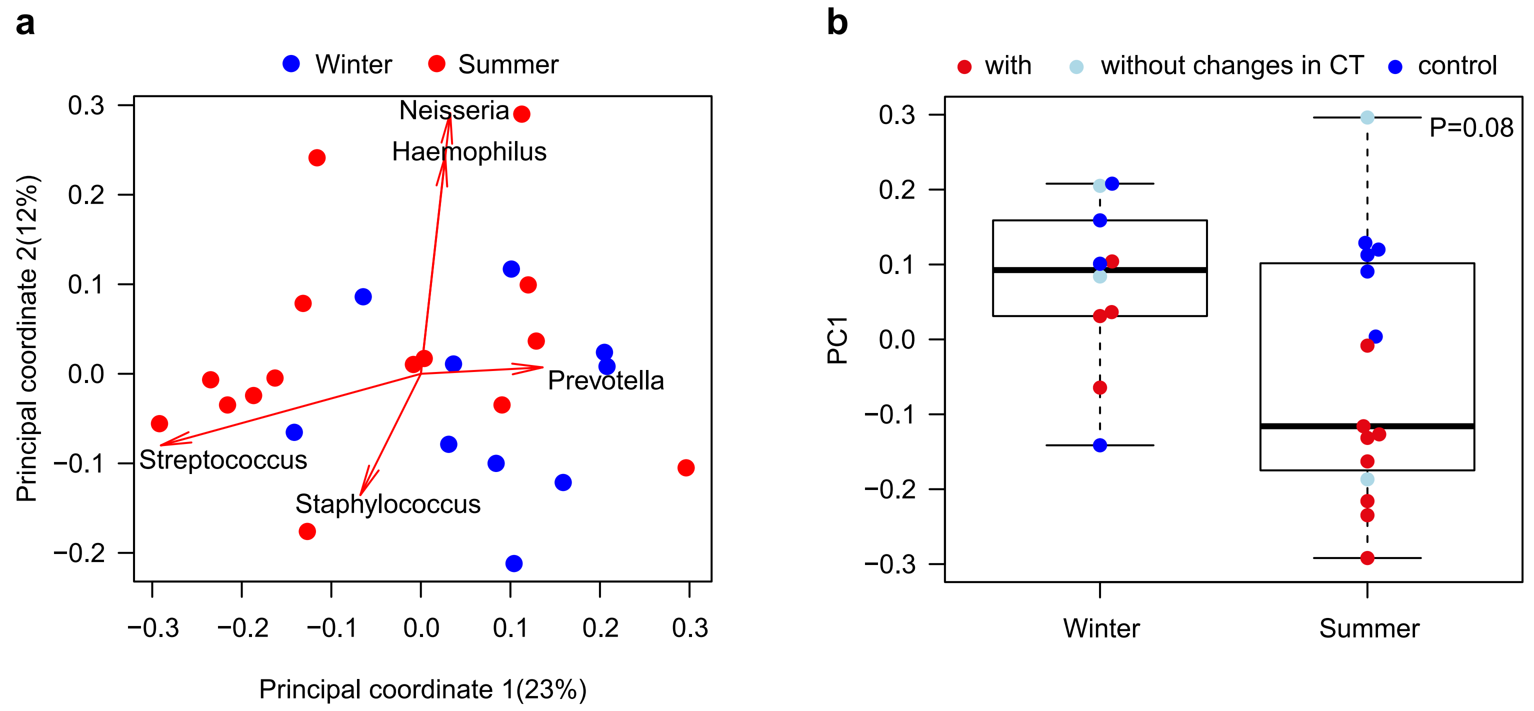

Figure S1 Genus level community composition for samples processed during summer and winter months based on PCoA. (a) Lung derived microbial community composition for samples processed during summer and winter months. (b) Boxplot comparing principal coordinate 1 between samples processed during summer and winter. The p-value was computed based on a two-sided Wilcoxon-Mann-Whitney test.
