# Supplementary material for: Influence of lung CT changes in chronic obstructive pulmonary disease (COPD) on the human lung microbiome
Source: PLoS One. 2017 Jul 13;12(7):e0180859. doi: 10.1371/journal.pone.0180859 (PMC5509234; doi:10.1371/journal.pone.0180859)
Supplement: S2 Fig — All samples were subsampled to 722 reads. Colors of the rarefaction curves indicate the sample types. The number of reads is shown on the x-axis and the corresponding number of OTUs on the y-axis. (PDF) [file pone.0180859.s003.pdf]

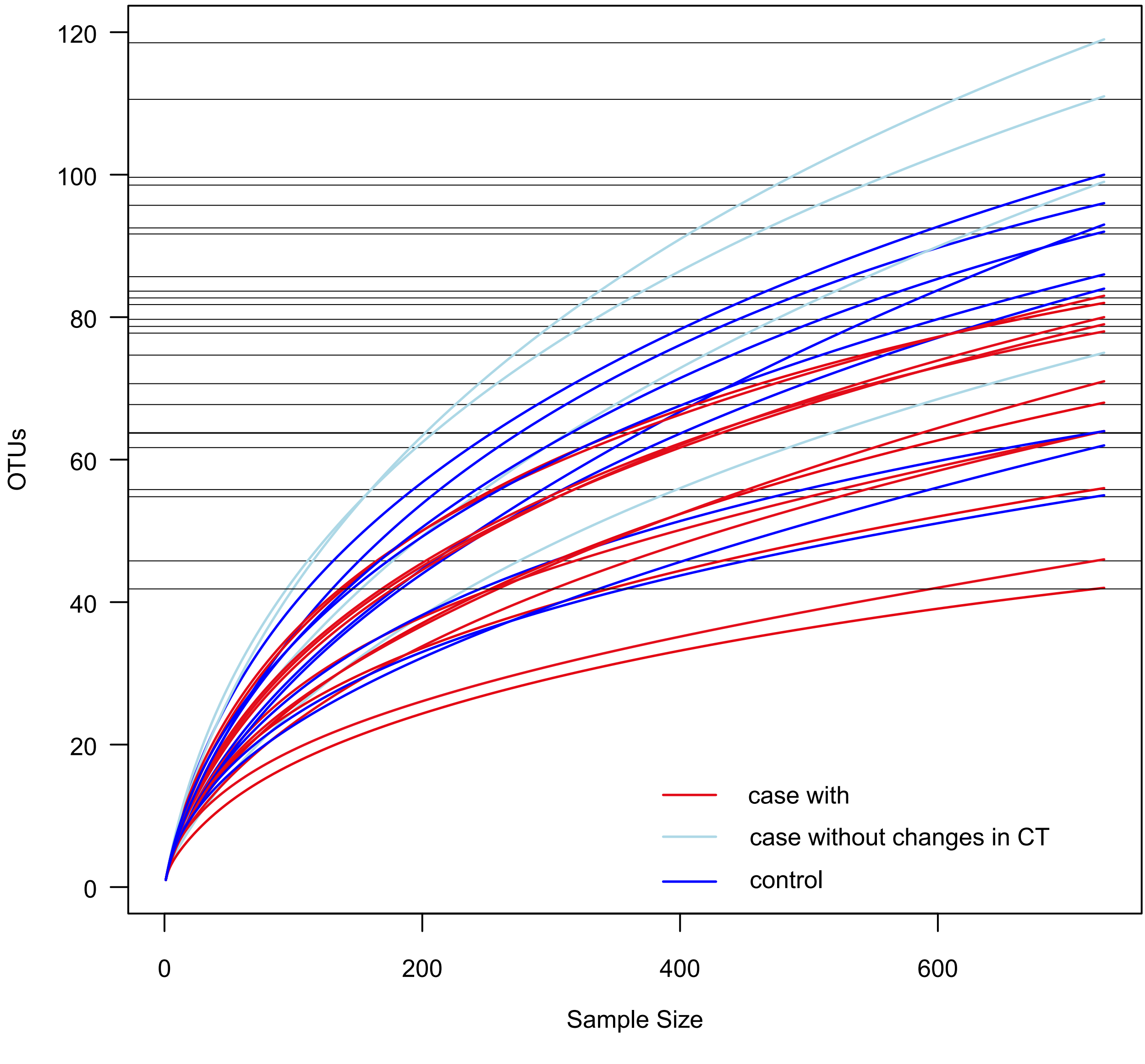

Figure S2 Rarefaction plot of COPD cases and controls for OTUs clustered on 95% identity. All samples were subsampled to 722 reads. Colors of the rarefaction curves indicate the sample types. The number of reads is shown on the x-axis and the corresponding number of OTUs on the y-axis.
