# Supplementary material for: Influence of lung CT changes in chronic obstructive pulmonary disease (COPD) on the human lung microbiome
Source: PLoS One. 2017 Jul 13;12(7):e0180859. doi: 10.1371/journal.pone.0180859 (PMC5509234; doi:10.1371/journal.pone.0180859)
Supplement: S3 Fig — (a) Lung derived microbial community composition for COPD patients with and without Glucocorticoid treatment. (b) Boxplot comparing principal coordinate 1 between COPD patients with and without Glucocorticoid treatment. The P-value was computed based on a two-sided Wilcoxon-Mann-Whitney test. (PDF) [file pone.0180859.s004.pdf]

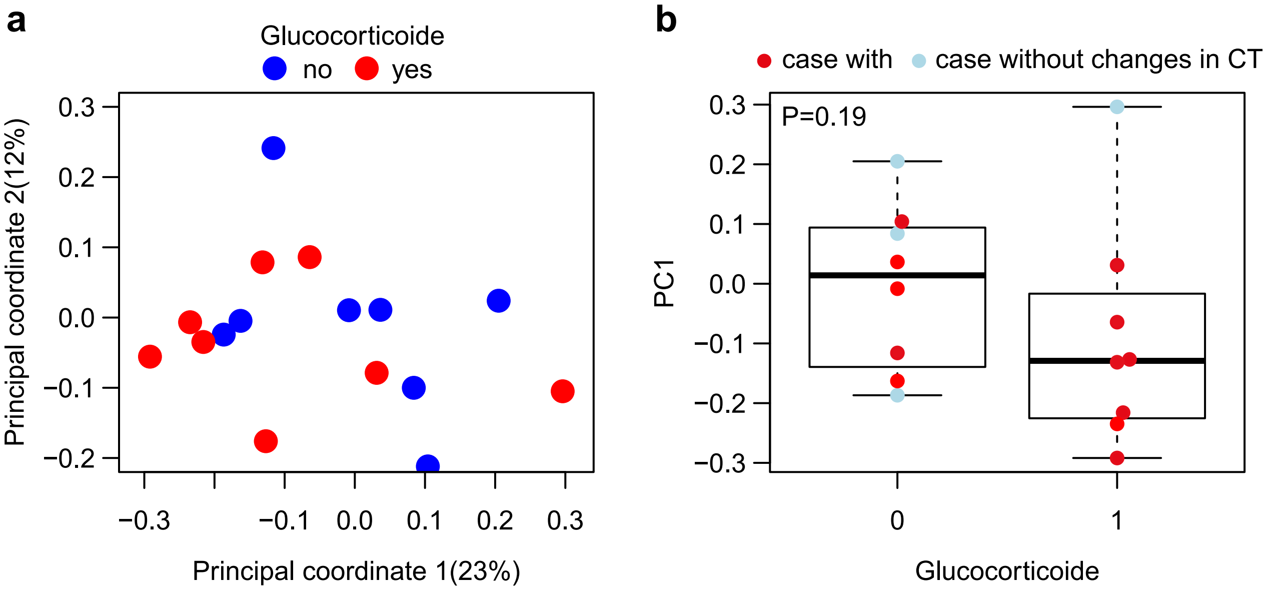

Figure S3 Genus level community composition for COPD patients with or without Glucocorticoid treatment. (**a**) Lung derived microbial community composition for COPD patients with and without Glucocorticoid treatment. (**b**) Boxplot comparing principal coordinate 1 between COPD patients with and without Glucocorticoid treatment. The p-value was computed based on a two-sided Wilcoxon-Mann-Whitney test.
