# Supplementary material for: Influence of lung CT changes in chronic obstructive pulmonary disease (COPD) on the human lung microbiome
Source: PLoS One. 2017 Jul 13;12(7):e0180859. doi: 10.1371/journal.pone.0180859 (PMC5509234; doi:10.1371/journal.pone.0180859)
Supplement: S4 Fig — Boxplots comparing principal coordinate 1 between severe subtype cases and controls P-value was computed based on two-sided Wilcoxon-Mann-Whitney tests after correction for confounding effects from samples processed during winter or summer months, respectively. (PDF) [file pone.0180859.s005.pdf]

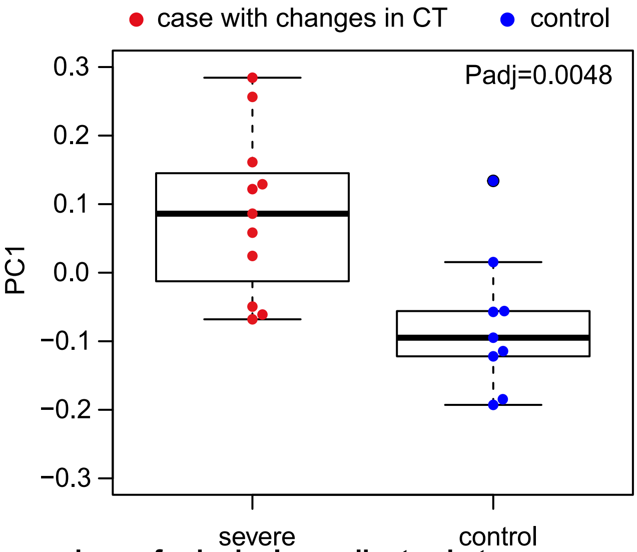

Figure S4 Comparison of principal coordinates between severe COPD subtypes and controls. Boxplots comparing principal coordinate 1 between control individuals and severe subtype patients
