# Supplementary material for: Influence of lung CT changes in chronic obstructive pulmonary disease (COPD) on the human lung microbiome
Source: PLoS One. 2017 Jul 13;12(7):e0180859. doi: 10.1371/journal.pone.0180859 (PMC5509234; doi:10.1371/journal.pone.0180859)
Supplement: S5 Fig — Boxplots show Hellinger transformed abundances for all genera that had significantly different abundances (P<0.05) in severe subtype versus mild subtype cases and controls. P-values were computed based on two-sided Wilcoxon-Mann-Whitney tests after correction for confounding effects from samples processed during winter or summer months, respectively. (PDF) [file pone.0180859.s006.pdf]

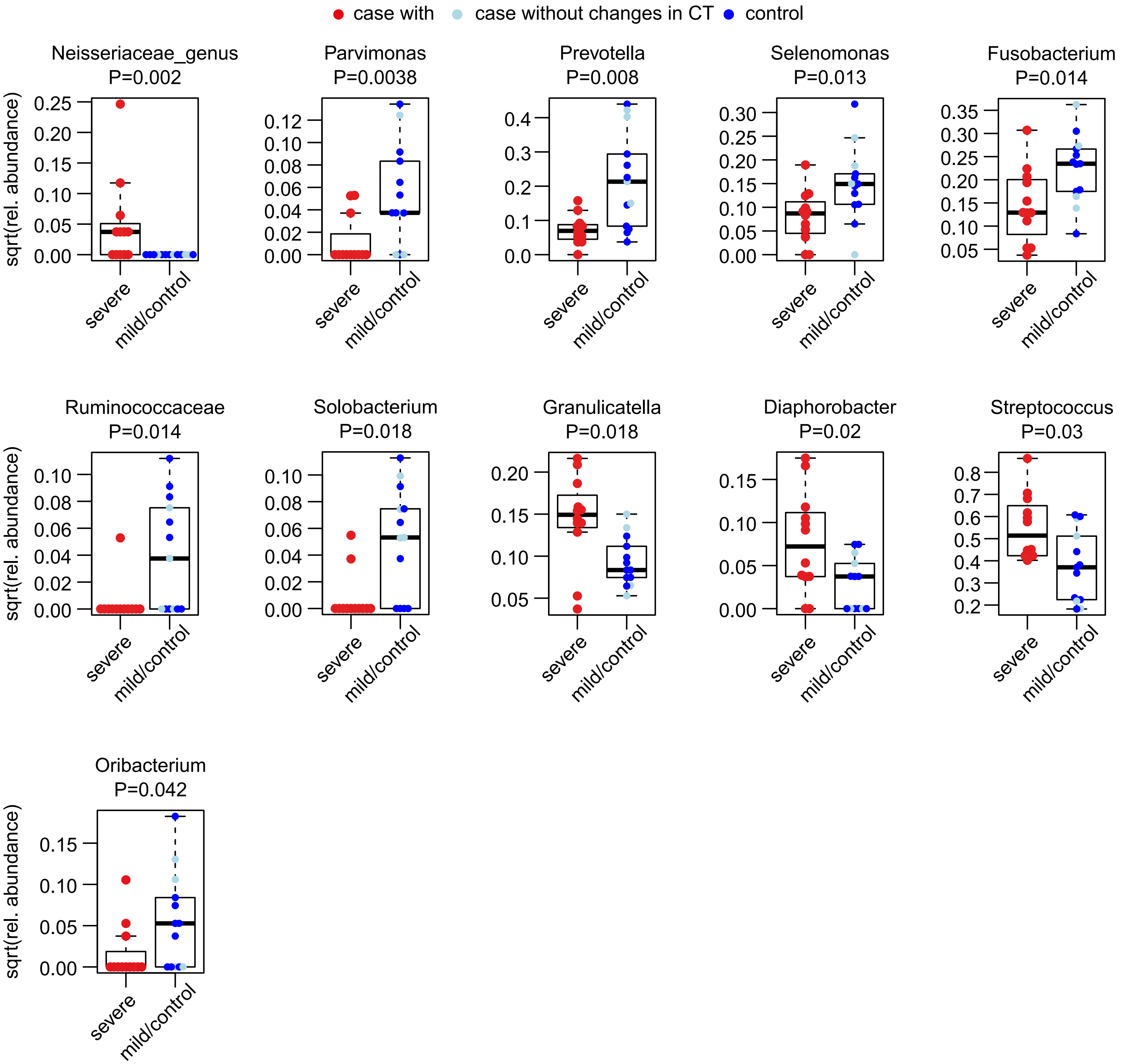

Figure S5 Genera with significantly different abundances between severe cases and mild cases and control individuals. Boxplots show Hellinger transformed abundances for all genera that had significantly different abundances (P<0.05) in severe cases compared to ae/control individuals. P-values were computed based on two-sided Wilcoxon-Mann-Whitney tests after correction for confounding effects from samples processed during winter or summer months, respectively.
