# Supplementary material for: Influence of lung CT changes in chronic obstructive pulmonary disease (COPD) on the human lung microbiome
Source: PLoS One. 2017 Jul 13;12(7):e0180859. doi: 10.1371/journal.pone.0180859 (PMC5509234; doi:10.1371/journal.pone.0180859)
Supplement: S7 Fig — Colors of nodes represent the different phyla and shading colors indicate microbial communities. (PDF) [file pone.0180859.s008.pdf]

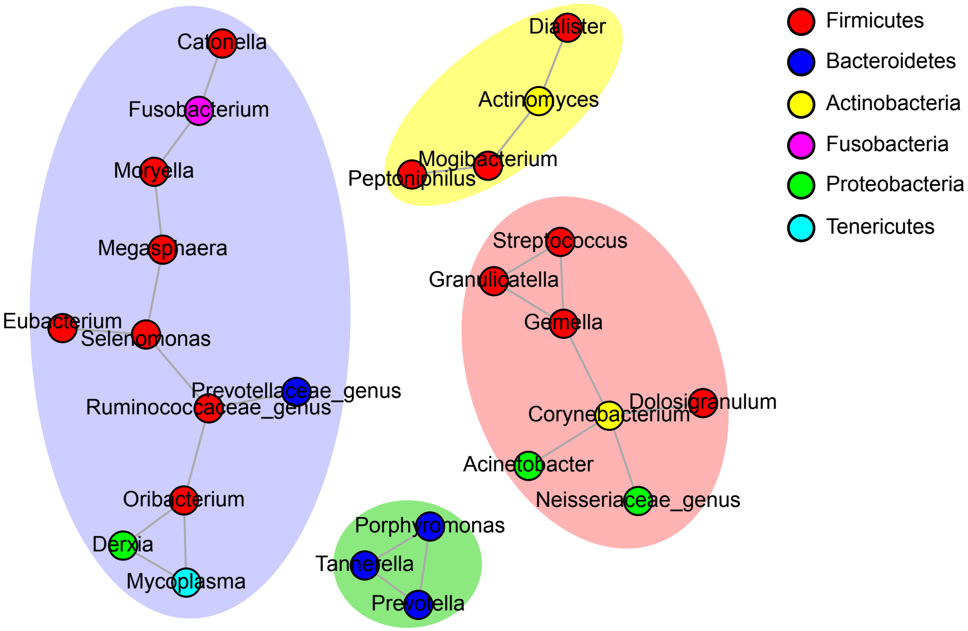

Figure S7 Phylum level composition of co-occurrence networks from bacterial genera. Colors of nodes represent the different phyla and shading colors indicate microbial communities
